# Causality of telomere length associated with calcific aortic valvular stenosis: A Mendelian randomization study

**DOI:** 10.3389/fmed.2022.1077686

**Published:** 2022-12-12

**Authors:** Junkui Wang, Yan Hao, Zhanfang Zhu, Bo Liu, Xuejun Zhang, Na Wei, Ting Wang, Ying Lv, Cuixiang Xu, Meijuan Ma, Yulian Zhang, Fuqiang Liu

**Affiliations:** ^1^Department of Cardiology, Shaanxi Provincial People’s Hospital, Xi’an, China; ^2^Shaanxi Provincial Key Laboratory of Infection and Immune Diseases, Shaanxi Provincial People’s Hospital, Xi’an, China; ^3^Xi’an Jiaotong University Hospital, Xi’an, China; ^4^Department of Nursing, Shaanxi Provincial People’s Hospital, Xi’an, China

**Keywords:** telomere length, calcific aortic valve stenosis, genome-wide association study, Mendelian randomization, single nucleotide polymorphism

## Abstract

**Background:**

Observational studies have shown that calcific aortic valve stenosis (CAVS) is associated with a shorter telomere length (TL). However, the results of observational studies are often influenced by confounding factors and reverse causal associations; it is unclear whether there is a causal relationship between TL and CAVS. This study aimed to investigate the causal relationship between TL and CAVS.

**Materials and methods:**

Genome-wide association study (GWAS) data on TL (*n* = 472,174) and CAVS (*n* = 311,437) were used to assess the effect of TL on CAVS. All the participants were of European ancestry. Three Mendelian randomization (MR) methods, namely, MR-Egger, weighted median, and inverse variance weighted (IVW), were used to assess the potential causal effect of TL on CAVS. Heterogeneity was assessed using Cochran’s *Q* statistic. Leave-one-out and MR-Egger regression methods were used for sensitivity and pleiotropy analyses. Forward and reverse MR analyses were performed.

**Results:**

In total, 118 valid and independent TL genetic instrumental variants were extracted from the GWAS dataset. MR analysis showed that TL was negatively associated with CAVS (odds ratios [OR] = 0.727, 95% confidence interval [CI]: 0.565–0.936, and *P* = 0.013 by weighted median; OR = 0.763, 95% CI: 0.634–0.920, and *P* = 0.005 by IVW; OR = 0.757, 95% CI: 0.549–1.044, and *P* = 0.055 by MR-Egger). Sensitivity and pleiotropy analyses showed that the results of this study were relatively stable and that there was no significant pleiotropy. Reverse MR analyses consistently suggested the absence of causal effects of CAVS liability on TL levels.

**Conclusion:**

A causal relationship between the shortening of TL and the development of CAVS in the European population was suggested in this study, and a theoretical basis was provided to investigate the pathogenesis of CAVS.

## Introduction

In developed countries, aortic valve stenosis (AVS) is the second most common cardiovascular disease after coronary artery disease and hypertension, and poses a serious health risk (1). The most common form of AVS is calcific aortic valve stenosis (CAVS), the incidence of which increases with age (2). The social burden caused by CAVS is expected to further increase in the coming decades due to the aging population and lack of effective preventive measures (3). Aortic valve replacement (AVR) is the only treatment option for severe CAVS; however, AVR is not suitable for all patients (4). CAVS has long been recognized as a degenerative disease associated with aging and aortic valve wear. Recently, several aging pathways associated with the development of CAVS have been identified, thus broadening ideas for the development of new CAVS diagnosis and treatment strategies (5).

Telomere length (TL) is an important marker of aging, which is influenced by both genetic and non-genetic factors. A shorter TL leads to cellular senescence and dysfunction, which, in turn, reduces the repair, and regeneration ability of cells (6). A growing number of studies have found that shorter TL is associated with various diseases, especially cardiovascular diseases (7). A meta-analysis showed that the risk of coronary heart disease increased by 54% when comparing the shortest and longest thirds of leukocyte TL (8). Genetic studies using single-nucleotide polymorphisms (SNPs) associated with TL have shown that individuals with genetic risk alleles of shorter TL have a higher risk of cardiovascular diseases (9). Saraieva et al. (10) found a shorter TL in calcified valve regions, suggesting that a shorter TL might be involved in the development of AVS, and the calcification process also seemed to promote a further local reduction of TL in the calcified region of the valve. However, a causal relationship between TL and CAVS has not been completely established.

Notably, traditional observational studies that are susceptible to confounding factors or reverse causality do not provide a causal relationship between exposure and outcome, which is susceptible to confounding factors or reverse causality (11). Randomized control trials (RCT) are the gold standard for clarifying causality; however, in reality, it is very difficult to complete an RCT, which requires significant human and material resources, and it is difficult to conduct an RCT owing to ethical issues (12). Mendelian randomization (MR) is a more robust method to reveal the causal link between an exposure and an outcome using genetic variants as instrumental variables. MR utilize instrumented genetic variation randomly distributed according to Mendel’s laws of inheritance during conception to mimic the randomization process of a randomized clinical trial, which overcome the limitations of observational cohort studies (13). In this study, we examined data from a genome-wide association study (GWAS) and aimed to performed a bi-directional MR study to investigate the causality of TL associated with CAVS.

## Materials and methods

### Data sources of telomere length and calcific aortic valvular stenosis

The summary statistics for TL were downloaded from the Integrative Epidemiology Unit (IEU) OpenGWAS Project Database^1^ by searching GWAS ID: ieu-b-4879, which mainly comprises publicly available GWAS summary data (14, 15). The data contained 472,174 samples of European ancestry from UK Biobank and 20,134,421 SNPs. The data were adjusted for covariates including age, sex, array, and the first ten principal components. CAVS GWAS data were obtained from the FinnGen Project Database,^2^ consisting of 9,153 patients with CAVS and 302,284 healthy controls of European ancestry (16). The FinnGen study is a public-private partnership project that combines genotype data from Finnish biobanks and digital health record data from Finnish health registries.

### Selection of genetic instrumental variants

The MR analysis must satisfy three assumptions: First, genetic instrumental variants are reliably associated with exposure variables. Second, genetic instrumental variants should not be associated with outcomes. Third, genetic instrumental variants strongly affect outcomes through exposure variables, but not through other pathways. We obtained a more accurate effect size estimate with these variants by removing three specific types of variants: (1) variants with linkage disequilibrium (LD) (*R*^2^ > 0.001), which were removed using LDlink (CEU);^3^ (2) palindromic variants resulting in potential strand ambiguity; and (3) variants identified as outliers by the MR-PRESSO test. We calculated the *F* statistics for the SNPs to evaluate instrument strength, where *F* statistics ≥10 indicated that the results did not suffer from weak instrument bias.

To select SNPs associated with TL as genetic instrument variants, we kept the threshold of statistical significance the same as before (*P* < 5 × 10^–8^, LD *r*^2^ < 0.1). After excluding SNPs associated with outcomes and confounding factors, we obtained 118 independent genetic SNPs associated with TL in this study (Supplementary Table 1). Similarly, 10 independent genetic SNPs were identified with genome-wide significance levels (*P* < 5 × 10^–8^) and were selected as genetic instruments for CAVS to perform a reverse MR analysis (Supplementary Table 2).

### Heterogeneity analysis

Heterogeneity may exist between the two samples according to the MR analysis owing to differences in the analysis platforms and enrollment populations. Therefore, in this study, a heterogeneity analysis was performed using the main inverse variance-weighted (IVW) and MR-Egger analysis methods based on Cochran’s *Q* statistic (17). *P* > 0.05 was considered as possessing no heterogeneity in the instrumental variants included. In addition, we visualized the heterogeneity of causal estimates using funnel plots.

### Mendelian randomization analysis

The TwoSampleMR R package was used to perform MR analysis. We selected three MR analysis methods: MR-Egger, weighted median, and IVW (18). Through the application of IVW, it was assumed that all SNPs were valid instrumental variants (19). The Wald ratio estimates were pooled using this method for individual SNPs using the inverse of the variance as weights. When at least 50% of the SNPs are valid instrumental variants, a summed estimate of the final effect is obtained *via* the weighted median (20). The MR-Egger method does not force the regression line through the origin, allowing directional genetic pleiotropy in the instrumental SNPs included (21). The results of the MR analysis were summarized in a tabular format, along with odds ratios (OR) and a confidence interval of 95% (CI) for all beta estimates. We generated scatter plots and trend lines associated with the different MR methods. The slopes and directions of the trend lines represent the magnitudes and directions of the causal estimates, respectively.

### Single single-nucleotide polymorphism analysis

The TwoSampleMR R package was used to analyze the effect of a single TL SNP on CAVS. The causal effects of all SNPs were estimated using IVW and MR-Egger pooling. The final results were presented as a forest plot.

### Sensitivity analysis

We assessed whether causal estimates were significantly affected by individual SNPs using the leave-one-out method (22), through which the combined effect of the remaining SNPs was separately calculated by eliminating them one by one, and the magnitude of the effect of each SNP on the results was observed. If the results of the leave-one-out analysis were inconsistent with those of the causal effect analysis, they indicated the presence of non-specific SNPs that may influence the causal estimation effect.

### Pleiotropy analysis

We assessed the magnitude of horizontal pleiotropy using the MR-Egger regression intercept, which can be used to evaluate the size of pleiotropy. The closer the intercept is to zero, the less likely it is that the gene is pleiotropic. *P* > 0.05, indicated that the likelihood of genetic pleiotropy in causal analysis was weak, and its effect could be ignored.

#### Power calculations

Statistical power was calculated using an online calculator for MR available at^4^ (23). Calculations were based on a type-one error rate of 0.05, the proportion of phenotypic variance explained by genetic variants (*R*^2^) for TL, and the total number of cases.

## Results

### Genetic variants selected as instrument variants

A total of 118 instrumental variables for TL were carefully selected to explore the effect of TL genetic instrument variants on CAVS, excluding 16 variants with LD and 20 palindromes (Supplementary Table 1). No outlier variants were identified using the MR-PRESSO outlier test. The *F*-statistic, reflecting the strength of the instruments, was 121.8, and which was larger than the generally selected value of 10, indicating no substantial weak instrument bias. All of the selected instruments collectively explained approximately 2.96% of the phenotypic variation in TL. Supplementary Table 3 shows detailed information on 118 TL genetic instrument variants in the CAVS GWAS dataset.

### Heterogeneity analysis

Heterogeneity analysis was performed using Cochran’s *Q* statistic. Table 1 shows the results of heterogeneity analysis of the 118 TL genetic instrument variants in the CAVS GWAS dataset. Statistically significant heterogeneity was observed in either the MR-Egger or IVF analysis (*P* < 0.05). However, the distribution of the genetic instrument variants in the funnel plot was relatively symmetrical (Supplementary Figure 1). Therefore, we used a random-effects model to estimate the MR effect size.

**TABLE 1:** Mendelian randomization (MR) analyses effect estimates for causal association of telomere length (TL) with risk of calcific aortic valvular stenosis (CAVS).

Methods	SNPs	Beta	SE	OR (95% CI)	*P*-value	Cochran’s *Q* statistic	*P*-value for Cochran’s Q	Egger_intercept	*P*-value for egger intercept	Power of MR
IVW	118	–0.298	0.092	0.742 (0.619–0.889)	0.001	182.76	5.94 × 10^–5^	0.0003	0.956	0.92
MR-Egger	118	–0.305	0.158	0.737 (0.541–1.004)	0.055					
Weight median	118	–0.319	0.121	0.727 (0.573–0.922)	0.008					

### Pleiotropy analysis

The MR-Egger intercept test was performed to determine pleiotropy. Statistical *P* = 0.956 suggests that there were no significant pleiotropic variants among the 118 selected TL genetic instrument variants in the CAVS GWAS datasets. These findings validated the hypothesis that genetic instrument variants are not associated with any confounding factors.

### Mendelian randomization analysis

**FIGURE 1:**
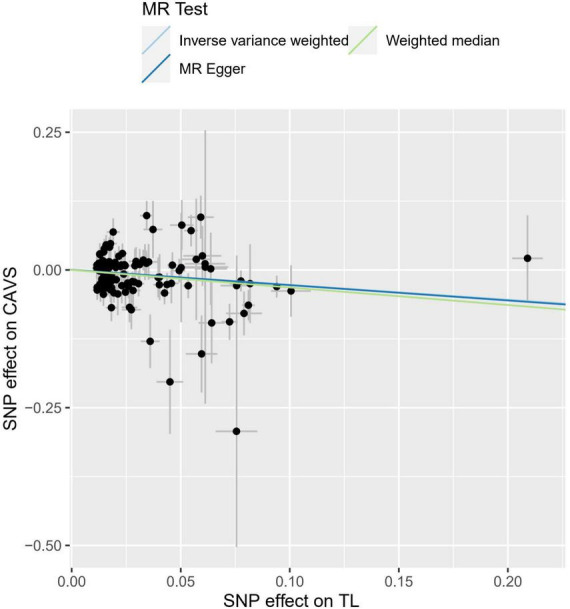
Individual estimates about the causal effect of telomere length (TL) on calcific aortic valve stenosis (CAVS). The *X*-axis shows the single nucleotide polymorphism (SNP) effect and standard error (SE) on each of the 118 TL SNPs from Integrative Epidemiology Unit (IEU) OpenGWAS dataset (https://gwas.mrcieu.ac.uk/datasets/ieu-b-4879). The *Y*-axis shows the SNP effect and SE on CAVS from FinnGen Project Database (https://r7.finngen.fi/). The regression lines for the MR-egger, weighted median, inverse variance weighted (IVW) method are shown. TL, telomere length; CAVS, calcific aortic valve stenosis; SNP, single nucleotide polymorphism; SE, standard error.

The OR values obtained through the weighted median and IVW methods were 0.742 (95% CI: 0.619–0.889, *P* = 0.001) and 0.727 (95% CI: 0.573–0.922, *P* = 0.008), respectively (Table 1), which suggested a negative causal relationship between TL and CAVS. The results from the MR-Egger method showed a consistent but non-significant trend (*P* = 0.055). As shown in Figure 1, scatter plots and trend lines indicate a negative causal relationship between TL and CAVS according to the different MR methods. The MR-Egger intercept did not deviate significantly from zero, thus validating the effect estimate. The MR power calculation yielded a result of 0.92, which suggested a strong ability to detect a significant causal effect of TL on CAVS.

### Single single-nucleotide polymorphism effect analysis

**FIGURE 2:**
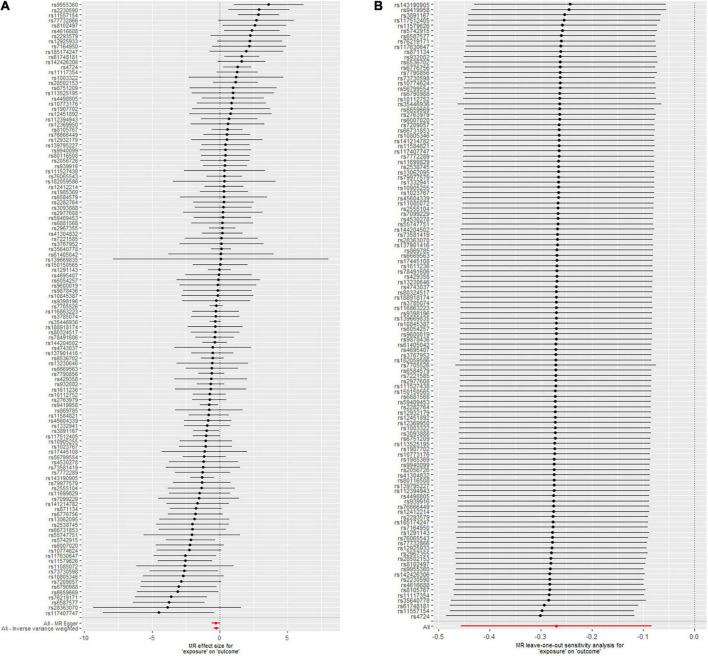
Mendelian randomization (MR) leave-one-out sensitivity analysis **(A)** and forest plot **(B)** of the 118 telomere length (TL) single nucleotide polymorphisms (SNPs) associated with risk of calcific aortic valve stenosis (CAVS).

The leave-one-out analysis of the effects of the 118 TL SNPs on CAVS is shown in Figure 2A. These findings indicate that our results are robust with no obvious bias based on the effect of a single TL SNP on CAVS. Figure 2B shows the effect of each TL SNP on CAVS, as well as the total causal effect obtained through the IVW and MR-Egger methods, which also revealed that the effect was not driven by a single SNP.

### Reverse mendelian randomization analysis

**FIGURE 3:**
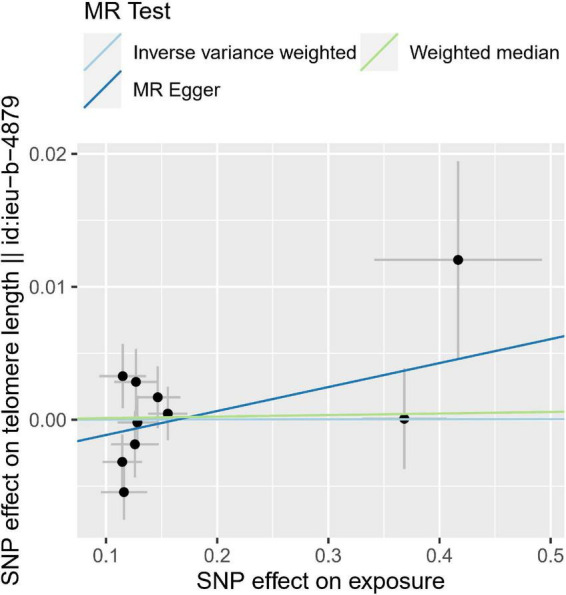
Individual estimates about the reverse causal effect of calcific aortic valve stenosis (CAVS) on telomere length (TL). The *X*-axis shows the single nucleotide polymorphism (SNP) effect and standard error (SE) on each of the 10 CAVS SNPs. The *Y*-axis shows the SNP effect and SE on TL. TL, telomere length; CAVS, calcific aortic valve stenosis; SNP, single nucleotide polymorphism; SE, standard error.

We then performed reverse MR analysis using CAVS-SNPs as IVs to test their effects on TL. We directly extracted the summarized statistics of 10 verified SNPs (*P* < 5.00 × 10^–08^) that related to CAVS. MR analysis using the IVW method showed that genetically predicted CAVS was not causally associated with TL (OR = 1.0009, 95% CI: 0.987–1.0132, *P* = 0.989) (see Figure 3 and Table 2). No indications of horizontal pleiotropy were detected using the MR-Egger intercept test (*P* > 0.1), and no outliers were detected in the MR-PRESSO analyses (Global Test *P* = 0.104).

**TABLE 2:** Mendelian randomization (MR) analyses effect estimates for association between calcific aortic valvular stenosis (CAVS) and risk of telomere length (TL).

Methods	SNPs	Beta	SE	OR (95% CI)	*P*-value	Cochran’s *Q* statistic	*P*-value for Cochran’s *Q*	Egger_ intercept	*P*-value for egger intercept
IVW	10	0.001	0.007	1.0009 (0.987–1.013)	0.989	16.12	0.064	0.0027	0.307
MR-Egger	10	0.018	0.017	1.018 (0.983–1.054)	0.339				
Weight median	10	0.001	0.006	1.001 (0.987–1.015)	0.863				

## Discussion

Using genetic variants as instrumental variants allows reasonable inference of a causal relationship between exposure and outcome (24). This study included 118 TL genetic instrumental variants. The IVW analysis showed that TL was negatively associated with the risk of CAVS, with consistent weighted median results. Sensitivity and multiplicity analyses emphasized the robustness of our findings, as well as the absence of horizontal pleiotropy and outliers in this study. Meanwhile, reverse MR analysis showed that genetically predicted CAVS did not affect TL levels. Our data provided evidence supporting the causal effect of TL on CAVS using the MR approach.

The development of CAVS has many similarities with that of atherosclerosis. Clinical studies have reported that individuals with shorter TL are prone to atherosclerotic cardiovascular disease (ASCVD) (25). Previous studies on MR have shown that alleles associated with a shorter TL are overrepresented in patients with ASCVD, suggesting a causal role for a shorter TL in the development of ASCVD (7). Saraieva et al. (10) suggested that a shorter TL might precede CAVS and render the valve more susceptible to the development of clinically significant AVS. In a recent animal model, Theodoris et al. found that TL shortening causes age-dependent premature aortic valve calcification *via* the RUNX2 pathway (26). Kurz et al. (27) hypothesized that a telomere-based cellular senescence program might be associated with the development of CAVS. Although their findings suggested a clear association between a shorter TL and CAVS, they were unable to demonstrate a causal relationship due to sample size or potential confounders. Since genetic variants are allocated randomly during conception which highly similar to RCTs, they may be exempt from confounding by other environmental exposures and reverse causation allowing uniquely reliable assessment of causal associations in MR analysis. To the best of our knowledge, this is the first large-scale MR analysis that comprehensively determines the causal relationship between TL and CAVS. Through MR analysis, a negative association between TL and CAVS was confirmed in our study, providing strong evidence for the causal relationship between TL and CAVS.

The mechanisms underlying the increased incidence of CAVS in relation to age are unclear; however, telomere-driven cellular senescence is of increasing interest. At the cellular level, senescence leads to a permanent non-dividing state that can induce changes in gene expression and cellular function (28). Telomeres shorten during somatic cell replication, which can eventually lead to cell cycle arrest and cell death if not repaired (29). Studies have shown that TL is shorter at sites of increased hemodynamic stress (30), suggesting that prolonged exposure to high mechanical and shear stress predisposes senescent cells to accumulate in the aortic valve, leading to the development of age-related CAVS. In addition, TL is influenced by genetic factors (31). Therefore, patients with inherited short telomeres may be more susceptible to CAVS. Zhan et al. (32) also suggested that the link between short telomeres and an increased risk of CAVS may be mediated through insulin-related pathways. Oxidative stress and inflammatory responses may also contribute to telomere shortening and are involved in the development of atherosclerosis in the cardiovascular system (33).

Our study has several advantages. First, our data were derived from the large-scale TL GWAS dataset (*n* = 472,174) and the largest CAVS GWAS dataset (*n* = 311,437) from public sources, so the strong instruments from large studies and the high *F*-statistics reduced the risk of weak instrument bias, and our result is transparent and reproducible. Second, the application of bidirectional MR design and different MR analysis methods were more robust to reduce confounding factors and reverse causation. Third, the sensitivity and pleiotropy analyses ensure the valid estimation of true MR causal effect size.

Our study has some limitations. First, although all participants included were from European ancestry (34, 35), the genetics of the Finnish population may differ from the other European populations, which lead to some biases based on ethnicity, so further generalization of this study to other populations is required. Second, since we only used summary statistics rather than the original individual measures, different standards of quality control, and selection may have affected the results. Third, although we demonstrated that a inherited shorter TL is associated with an increased risk of CAVS, the exact mechanism remains unclear. However, the present study revealed a causal effect of TL on CAVS, thus providing new insights into the pathogenesis of CAVS.

## Conclusion

A causal relationship between TL and CAVS was suggested in this study; the primary conclusion of this study is that telomere biology, especially inherited short TL, is potentially involved in the development of CAVS, which helps guide therapeutic interventions.

## Data availability statement

The data supporting the findings of this work is available in the IEU OpenGWAS Project (GWAS ID: ieu-b-4879) at https://gwas.mrcieu.ac.uk/, and FinnGen Project Database (ID: I9_CAVS) at https://r7.finngen.fi/.

## Ethics statement

The present study was based on publicly available summary-level data in the Integrative Epidemiology Unit (IEU) GWAS database and FinnGen consortium. Therefore, ethical approval was not necessary.

## Author contributions

JW and YH wrote the first draft of the manuscript. ZZ, YZ, and FL developed the research question. BL, XZ, NW, TW, YL, CX, and MM contributed to the development of the review protocol, data analysis, and refining of the manuscript, and approved the final manuscript. YZ and FL critically read and revised the manuscript before submission. All authors have read and approved the final manuscript.

## Footnotes

1
https://gwas.mrcieu.ac.uk/

2
https://r7.finngen.fi/

3
https://ldlink.nci.nih.gov/?tab=ldmatrix

4
https://shiny.cnsgenomics.com/mRnd/

## Supplementary material

Supplementary Figure 1 Funnel plot to visualize overall heterogeneity of Mendelian randomization (MR) estimates for the effect of telomere length (TL) on calcific aortic valve stenosis (CAVS).

Supplementary Table 1 Summary statistics of the telomere length (TL) genetic instrumental variables (IVs).

Supplementary Table 2 Summary statistics of the calcific aortic valve stenosis (CAVS) genetic instrumental variables (IVs).

Supplementary Table 3 Association of the telomere length (TL) genetic instrumental variables (IVs) with calcific aortic valve stenosis (CAVS) genome-wide association study (GWAS).
