# Supplementary figures and images for: Causality of telomere length associated with calcific aortic valvular stenosis: A Mendelian randomization study
Source: Front Med (Lausanne). 2022 Dec 12;9:1077686. doi: 10.3389/fmed.2022.1077686 (PMC9790894; doi:10.3389/fmed.2022.1077686)

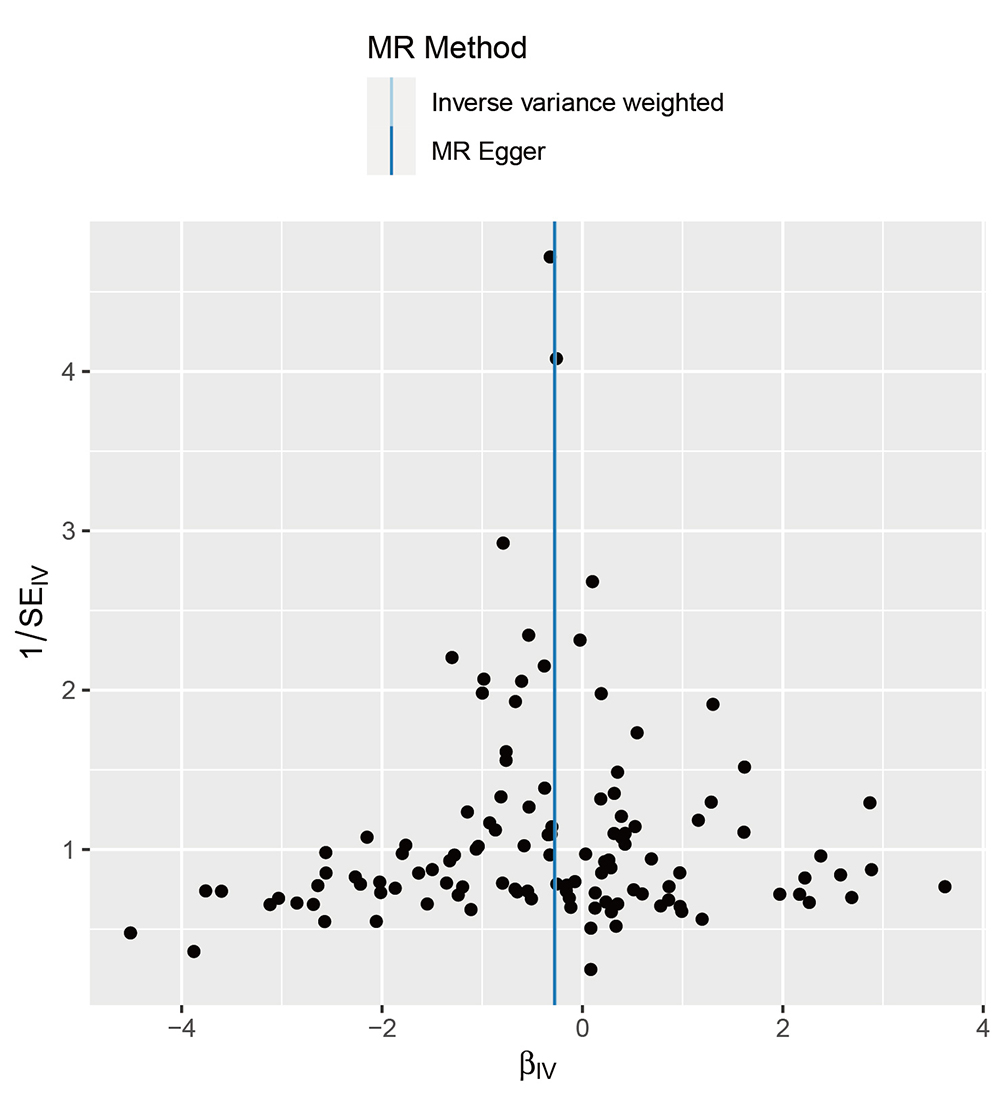

Supplement: Supplementary Figure 1 — Funnel plot to visualize overall heterogeneity of Mendelian randomization (MR) estimates for the effect of telomere length (TL) on calcific aortic valve stenosis (CAVS). [file Image_1.JPEG]
