# Supplementary material for: Causality of telomere length associated with calcific aortic valvular stenosis: A Mendelian randomization study
Source: Front Med (Lausanne). 2022 Dec 12;9:1077686. doi: 10.3389/fmed.2022.1077686 (PMC9790894; doi:10.3389/fmed.2022.1077686)
Supplement: Supplementary Table 1 — Summary statistics of the telomere length (TL) genetic instrumental variables (IVs). [file Table_1.pdf]

Supplementary Table 1 Summary statistics of the telomere length genetic instrumental variables (IVs)

SNP	EA	NEA	Genes	EAF	Beta	SE	p val	$R^{2\,(\%)}$	F
rs1003322	A	C	ARSA	0.214	0.014	0.0025	1.03E-08	0.0068	32.78
rs10112752	A	G	TERF1	0.43	-0.029	0.002	9.51E-46	0.0405	201.56
rs1023767	A	G	KIAA1429	0.238	-0.018	0.0023	5.04E-15	0.0122	61.25
rs10773176	G	A	ZCCHC8	0.741	-0.017	0.0023	5.21E-14	0.0114	56.65
rs10774624	A	G	SH2B3	0.533	0.015	0.0021	2.95E-13	0.0112	53.24
rs10805346	C	T	SLC2A9	0.439	0.012	0.002	6.98E-09	0.0068	33.54
rs10845387	A	G	ETV6	0.353	-0.014	0.0021	1.54E-11	0.0091	45.48
rs10905255	T	G	GDI2	0.579	-0.018	0.002	2.58E-19	0.0162	80.74
rs11085072	T	C	SH3GL1	0.237	-0.013	0.0024	2.57E-08	0.0063	31
rs11117354	C	T	BANP	0.697	0.023	0.0022	3.40E-26	0.0229	112.1
rs111527438	C	T	ADAP2	0.351	0.013	0.0021	3.15E-09	0.0071	35.09
rs112394943	C	T	LMLN	0.163	-0.02	0.0028	1.61E-12	0.0108	49.91
rs113525195	A	C	PSMB5	0.29	-0.012	0.0022	3.10E-08	0.0063	30.65
rs11557154	T	C	DCAF12	0.13	-0.034	0.003	1.13E-30	0.0267	132.56
rs11579626	C	A	CHD1L	0.085	0.027	0.0036	1.26E-13	0.0109	54.92
rs11584821	T	C	BCL2L15	0.176	-0.031	0.0026	3.00E-31	0.0273	135.19
rs116863223	A	G	ENOSF1	0.012	-0.082	0.0094	2.61E-18	0.0156	76.16
rs11699829	A	G	PTK6	0.034	0.064	0.006	1.51E-26	0.0272	113.7
rs117407747	T	C	VIPR2	0.028	0.045	0.0061	1.77E-13	0.0109	54.25
rs117512405	A	G	UCKL1	0.017	-0.079	0.0082	9.53E-22	0.0209	91.81
rs117630647	A	G	POT1	0.021	0.06	0.0072	1.36E-16	0.0148	68.36
rs12369950	C	T	BCAT1	0.141	-0.018	0.0029	8.04E-10	0.0077	37.75
rs12412214	A	G	NKX2-3	0.28	-0.025	0.0022	3.42E-28	0.0242	121.22
rs12451892	C	T	SGSM2	0.381	-0.012	0.0021	2.20E-08	0.0064	31.31
rs1291143	C	A	SAMHD1	0.849	0.049	0.0028	1.79E-69	0.0623	310.39
rs12925933	C	A	PRDM7	0.662	-0.015	0.0021	6.98E-12	0.0096	47.03
rs12932179	G	A	USP7	0.561	-0.014	0.002	1.82E-11	0.0091	45.16
rs13062095	C	T	TRMT10C	0.328	0.014	0.0021	9.74E-11	0.0085	41.87
rs13230646	C	T	STK31	0.249	-0.017	0.0023	8.87E-14	0.0112	55.6
rs1332941	G	A	KBTBD6	0.82	0.026	0.0027	5.88E-21	0.0194	88.21
rs137901416	A	G	DCAF4	0.1	0.046	0.0033	4.66E-43	0.0377	189.24
rs139669835	T	C	YES1	0.009	-0.061	0.0105	6.07E-09	0.007	33.81
rs139795227	C	A	RPAP2	0.014	0.06	0.0087	6.71E-12	0.0099	47.11
rs141214782	C	T	PAPD4	0.101	-0.025	0.0034	2.01E-13	0.0111	53.99
rs142426306	T	C	ABHD16B	0.04	-0.05	0.0054	8.66E-21	0.0194	87.45
rs143190905	T	G	RTEL1	0.08	-0.072	0.0037	1.61E-85	0.0775	384.09
rs144204502	T	C	TK1	0.013	-0.101	0.0091	3.37E-28	0.0251	121.25
rs150150565	T	C	ENOSF1	0.021	0.064	0.0074	6.82E-18	0.0171	74.27
rs1611236	A	G	HLA-F	0.327	-0.016	0.0021	6.12E-14	0.0113	56.33
rs17445108	A	G	PTGES3	0.127	-0.017	0.003	2.00E-08	0.0063	31.5
rs182059586	C	T	PARN	0.025	-0.057	0.0068	4.91E-17	0.016	70.37
rs185174247	A	G	UBE2D2	0.056	0.037	0.0044	1.06E-17	0.0147	73.4
rs188918174	T	C	ACYP2	0.036	0.04	0.0054	1.22E-13	0.0113	54.98
rs1907702	A	G	KITLG	0.767	0.015	0.0024	5.94E-10	0.0081	38.34
rs1985369	G	A	VIPR2	0.868	-0.031	0.003	3.63E-25	0.0223	107.4
rs2056726	A	G	STAG3	0.214	-0.023	0.0024	7.87E-21	0.0175	87.63
rs2230590	C	T	MST1R	0.511	-0.016	0.002	3.56E-15	0.0125	61.93

rs2282764	G	A	MXD4	0.142	-0.022	0.0029	9.30E-15	0.0123	60.04
rs2293579	A	G	PSMC3	0.386	-0.013	0.0021	3.27E-10	0.0079	39.5
rs2538745	C	T	POMZP3	0.603	-0.013	0.0021	3.08E-10	0.008	39.62
rs2555104	C	A	VSNL1	0.434	-0.014	0.002	6.61E-12	0.0096	47.14
rs2763979	T	C	HSPA1B	0.36	-0.028	0.0021	1.26E-40	0.0355	178.11
rs28363070	A	G	SLC6A3	0.013	0.076	0.0096	3.53E-15	0.0151	61.94
rs28502153	A	C	GAB4	0.378	-0.022	0.0021	1.18E-25	0.0219	109.64
rs2967355	C	A	MPHOSPH6	0.774	-0.046	0.0024	3.95E-83	0.0745	373.1
rs2977608	C	A	SAMD11	0.744	0.013	0.0023	3.02E-08	0.0064	30.69
rs3093888	A	G	PARP2	0.051	-0.029	0.0045	1.52E-10	0.0082	41
rs35446936	A	G	ACTRT3	0.244	-0.094	0.0023	3.56E-198	0.3257	1628.82
rs35640778	A	G	RTEL1	0.021	-0.209	0.007	9.57E-195	0.1776	886.25
rs3767952	A	G	NFYC	0.227	0.013	0.0024	1.80E-08	0.0063	31.7
rs3785074	G	A	TERF2	0.29	0.024	0.0022	2.64E-27	0.0234	117.17
rs3891167	G	A	TYMS	0.253	-0.043	0.0024	1.20E-70	0.0686	315.78
rs41304832	A	G	ZBTB46	0.012	0.061	0.0093	5.01E-11	0.0091	43.17
rs429358	C	T	APOE	0.154	0.017	0.0028	3.82E-10	0.0078	39.21
rs4498805	T	G	SLC16A4	0.547	0.015	0.002	5.65E-14	0.0112	56.49
rs4530278	T	G	SLC7A10	0.598	0.014	0.0021	1.50E-11	0.0093	45.54
rs45604339	T	C	MAX	0.342	-0.02	0.0021	4.29E-22	0.0188	93.39
rs4616688	T	G	IFT80	0.525	-0.017	0.002	4.51E-18	0.015	75.09
rs4695407	G	A	OCIAD1	0.508	0.014	0.002	1.46E-12	0.01	50.1
rs4724	A	G	NAA38	0.117	-0.055	0.0031	9.81E-69	0.0617	307.01
rs4743037	T	C	ZNF462	0.231	0.015	0.0024	5.14E-10	0.0078	38.62
rs55747751	A	G	HSPA4	0.077	-0.021	0.0038	1.70E-08	0.0064	31.82
rs56799554	G	A	ARL4D	0.17	-0.026	0.0027	3.05E-22	0.0191	94.07
rs5742915	C	T	PML	0.446	0.019	0.002	1.55E-21	0.0185	90.85
rs59409453	G	A	SERPINF1	0.731	0.02	0.0023	1.61E-18	0.0161	77.12
rs6007020	C	T	SMC1B	0.368	0.014	0.0021	4.77E-12	0.0098	47.78
rs6054257	A	G	DEFB125	0.794	-0.014	0.0025	1.07E-08	0.0066	32.71
rs61405042	T	C	FAM20C	0.029	-0.05	0.006	8.54E-17	0.0143	69.28
rs61748181	T	C	TERT	0.029	-0.059	0.006	2.79E-23	0.0197	98.8
rs6536702	A	G	NAF1	0.775	0.053	0.0024	9.44E-111	0.0996	500.01
rs6584579	G	A	OBFC1	0.399	0.011	0.002	1.97E-08	0.0063	31.53
rs6587577	G	A	POGZ	0.826	-0.018	0.0026	4.84E-12	0.0095	47.75
rs6659669	T	C	IVNS1ABP	0.605	-0.012	0.0021	1.15E-08	0.0066	32.57
rs6669563	A	G	SPOCD1	0.438	0.018	0.002	2.13E-19	0.0164	81.12
rs66731853	A	G	CDA	0.317	-0.018	0.0022	1.54E-16	0.0137	68.12
rs6751209	C	T	THADA	0.204	-0.014	0.0025	1.57E-08	0.0064	31.96
rs6776756	A	G	GATA2	0.598	-0.017	0.002	1.11E-17	0.0146	73.3
rs6790988	G	A	CLDN11	0.742	0.015	0.0023	1.78E-10	0.0081	40.7
rs6881568	A	C	MRPL36	0.363	0.017	0.0021	3.71E-16	0.0132	66.38
rs7099229	A	G	NOC3L	0.273	-0.015	0.0022	8.44E-12	0.0093	46.66
rs7164950	G	A	MNS1	0.406	0.013	0.002	2.28E-10	0.0081	40.21
rs7209057	A	G	NOL11	0.561	0.012	0.002	5.68E-09	0.0069	33.94
rs7221585	T	C	AFMID	0.224	0.014	0.0025	6.65E-09	0.0071	33.63
rs73581419	T	C	RAB2B	0.107	0.023	0.0032	1.34E-12	0.0101	50.27
rs73730598	A	G	PLEKHG4B	0.055	0.027	0.0044	4.69E-10	0.0078	38.8
rs76065543	T	C	RFWD3	0.138	0.034	0.0029	4.22E-32	0.0279	139.08

rs76219171	A	G	PAPD5	0.058	0.036	0.0043	7.78E-17	0.0142	69.47
rs76666449	C	T	SRSF9	0.101	0.03	0.0033	8.17E-19	0.0158	78.46
rs7705526	A	C	TERT	0.327	0.078	0.0022	2.43E-282	0.2649	1289.26
rs7772289	T	G	ZBED9	0.503	0.018	0.002	1.72E-18	0.0154	76.99
rs77732866	A	G	FANCL	0.138	0.018	0.0029	9.16E-10	0.0075	37.5
rs7790856	T	C	POT1	0.289	-0.044	0.0022	1.80E-87	0.0786	393.04
rs78491606	C	A	SHQ1	0.018	-0.076	0.0074	1.90E-24	0.0207	104.13
rs79977579	A	C	NFE2	0.096	0.028	0.0034	2.34E-16	0.0137	67.29
rs80116508	A	G	SLX4	0.062	-0.035	0.0042	1.98E-17	0.0145	72.17
rs80324517	A	G	DUSP22	0.048	0.04	0.0047	1.84E-17	0.0144	72.31
rs8102497	A	G	ZIM2	0.432	-0.015	0.002	1.40E-13	0.011	54.71
rs8105767	G	A	ZNF257	0.295	0.033	0.0022	2.49E-50	0.0448	222.56
rs869785	C	T	THRB	0.672	-0.015	0.0021	4.45E-12	0.0096	47.92
rs871134	T	C	CCDC96	0.569	-0.018	0.002	1.71E-19	0.0164	81.55
rs932002	T	C	PARP1	0.151	-0.04	0.0028	7.31E-47	0.0414	206.67
rs9398196	G	A	CD164	0.52	-0.014	0.002	9.51E-13	0.0103	50.94
rs939916	A	G	BET1L	0.67	0.024	0.0022	6.63E-29	0.0259	124.47
rs9419958	C	T	OBFC1	0.861	-0.081	0.0029	2.64E-167	0.1567	760.03
rs9600019	T	C	BORA	0.336	0.013	0.0021	2.43E-09	0.0072	35.59
rs9878436	T	C	CEP70	0.434	-0.014	0.002	1.20E-12	0.0101	50.49
rs9940099	T	G	NLRC3	0.063	-0.034	0.0041	3.21E-16	0.0133	66.67
rs9955360	A	C	PARD6G	0.869	-0.019	0.003	2.18E-10	0.0082	40.3

SNP, single-nucleotide polymorphism; EA, effect allele; NEA, non-effect allele; EAF, effect allele frequency; Beta, the regression coefficient based on the telomere length effect allele; SE, standard error
