# Supplementary material for: Causality of telomere length associated with calcific aortic valvular stenosis: A Mendelian randomization study
Source: Front Med (Lausanne). 2022 Dec 12;9:1077686. doi: 10.3389/fmed.2022.1077686 (PMC9790894; doi:10.3389/fmed.2022.1077686)
Supplement: Supplementary Table 2 — Summary statistics of the calcific aortic valve stenosis (CAVS) genetic instrumental variables (IVs). [file Table_2.pdf]

Supplementary Table 2 Summary statistics of the calcific aortic valve stenosis genetic instrumental variables (IVs)

SNP	EA	NEA	EAF	Beta	SE	p val	$R^{2\,(\%)}$	F
rs11166276	T	C	0.483	0.156	0.0177	1.33E-18	0.0121	77.5
rs507068	G	A	0.419	0.128	0.0178	5.89E-13	0.008	51.88
rs309306	T	C	0.438	-0.125	0.0179	2.30E-12	0.0077	49.21
rs72854462	G	A	0.267	-0.146	0.0204	7.52E-13	0.0084	51.4
rs13121593	G	T	0.249	-0.116	0.0208	2.38E-08	0.005	31.16
rs117733303	G	A	0.011	0.417	0.0755	3.45E-08	0.0038	30.43
rs74617384	T	A	0.046	0.368	0.0379	3.15E-22	0.012	94
rs1800797	G	A	0.473	-0.114	0.0177	9.85E-11	0.0065	41.85
rs10770612	G	A	0.237	-0.126	0.0215	4.89E-09	0.0057	34.23
rs2869553	G	T	0.265	0.127	0.0196	1.04E-10	0.0063	41.74

SNP, single-nucleotide polymorphism; EA, effect allele; NEA, non-effect allele; EAF, effect allele frequency; Beta, the regression coefficient based on the telomere length effect allele; SE, standard error
