# Supplementary material for: Causality of telomere length associated with calcific aortic valvular stenosis: A Mendelian randomization study
Source: Front Med (Lausanne). 2022 Dec 12;9:1077686. doi: 10.3389/fmed.2022.1077686 (PMC9790894; doi:10.3389/fmed.2022.1077686)
Supplement: Supplementary Table 3 — Association of the telomere length (TL) genetic instrumental variables (IVs) with calcific aortic valve stenosis (CAVS) genome-wide association study (GWAS). [file Table_3.pdf]

Supplementary Table 3 Association of the telomere length genetic IVs with CAVS GWAS

SNP	TL			CAVS		
	Beta	SE	p val	Beta	SE	p val
rs1003322	0.014	0.0025	1.03E-08	0.0169	0.0252	0.5017
rs10112752	-0.029	0.002	9.51E-46	0.0219	0.0178	0.2197
rs1023767	-0.018	0.0023	5.04E-15	0.0195	0.0183	0.2885
rs10773176	-0.017	0.0023	5.21E-14	-0.0149	0.0224	0.5072
rs10774624	0.015	0.0021	2.95E-13	-0.034	0.0181	0.0609
rs10805346	0.012	0.002	6.98E-09	-0.0314	0.0179	0.0787
rs10845387	-0.014	0.0021	1.54E-11	0.0023	0.0191	0.906
rs10905255	-0.018	0.002	2.58E-19	0.019	0.0179	0.2897
rs11085072	-0.013	0.0024	2.57E-08	0.0339	0.0241	0.1594
rs11117354	0.023	0.0022	3.40E-26	0.0299	0.0179	0.0953
rs111527438	0.013	0.0021	3.15E-09	0.0044	0.019	0.8167
rs112394943	-0.02	0.0028	1.61E-12	-0.0137	0.0212	0.5168
rs113525195	-0.012	0.0022	3.10E-08	-0.0121	0.0193	0.5309
rs11557154	-0.034	0.003	1.13E-30	-0.0679	0.027	0.0121
rs11579626	0.027	0.0036	1.26E-13	0.0249	0.023	0.28
rs11584821	-0.031	0.0026	3.00E-31	0.0249	0.0716	0.7284
rs116863223	-0.082	0.0094	2.61E-18	-0.0961	0.0735	0.1909
rs11699829	0.064	0.006	1.51E-26	-0.2031	0.0946	0.0318
rs117407747	0.045	0.0061	1.77E-13	0.0788	0.0399	0.048
rs117512405	-0.079	0.0082	9.53E-22	-0.1524	0.0699	0.0293
rs117630647	0.06	0.0072	1.36E-16	-0.0107	0.0247	0.6666
rs12369950	-0.018	0.0029	8.04E-10	-0.0078	0.0181	0.6667
rs12412214	-0.025	0.0022	3.42E-28	-0.0091	0.018	0.6138
rs12451892	-0.012	0.0021	2.20E-08	-0.0011	0.0213	0.9576
rs1291143	0.049	0.0028	1.79E-69	-0.0326	0.0179	0.0683
rs12925933	-0.015	0.0021	6.98E-12	-0.007	0.0182	0.7022
rs12932179	-0.014	0.002	1.82E-11	-0.0259	0.0183	0.1576
rs13062095	0.014	0.0021	9.74E-11	0.0095	0.0235	0.6868
rs13230646	-0.017	0.0023	8.87E-14	-0.0237	0.022	0.2807
rs1332941	0.026	0.0027	5.88E-21	-0.0243	0.0361	0.5004
rs137901416	0.046	0.0033	4.66E-43	-0.0052	0.2483	0.9834
rs139669835	-0.061	0.0105	6.07E-09	0.0256	0.0544	0.6385
rs139795227	0.06	0.0087	6.71E-12	0.0403	0.029	0.1641
rs141214782	-0.025	0.0034	2.01E-13	-0.0814	0.0456	0.0739
rs142426306	-0.05	0.0054	8.66E-21	0.0942	0.0328	0.0041
rs143190905	-0.072	0.0037	1.61E-85	0.0382	0.0468	0.4141
rs144204502	-0.101	0.0091	3.37E-28	0.0021	0.0657	0.9746
rs145114957	0.064	0.0074	6.82E-18	0.0108	0.0213	0.6142
rs1611236	-0.016	0.0021	6.12E-14	0.0188	0.0271	0.489
rs17445108	-0.017	0.003	2.00E-08	-0.0192	0.1102	0.8615
rs182059586	-0.057	0.0068	4.91E-17	0.0735	0.0519	0.1566
rs185174247	0.037	0.0044	1.06E-17	-0.0131	0.0417	0.7545
rs188918174	0.04	0.0054	1.22E-13	0.0129	0.022	0.5565
rs1907702	0.015	0.0024	5.94E-10	-0.0099	0.0284	0.7277
rs1985369	-0.031	0.003	3.63E-25	-0.009	0.0212	0.6728

rs2056726	-0.023	0.0024	7.87E-21	-0.0456	0.0181	0.0118
rs2230590	-0.016	0.002	3.56E-15	-0.0064	0.0253	0.8008
rs2282764	-0.022	0.0029	9.30E-15	-0.0293	0.0194	0.1307
rs2293579	-0.013	0.0021	3.27E-10	0.026	0.0177	0.1422
rs2538745	-0.013	0.0021	3.08E-10	0.0189	0.0177	0.2849
rs2555104	-0.014	0.002	6.61E-12	0.0211	0.0178	0.2354
rs2763979	-0.028	0.0021	1.26E-40	-0.2929	0.2101	0.1633
rs28363070	0.076	0.0096	3.53E-15	-0.025	0.0182	0.1706
rs28502153	-0.022	0.0021	1.18E-25	-0.0087	0.0233	0.7092
rs2967355	-0.046	0.0024	3.95E-83	0.003	0.0193	0.8746
rs2977608	0.013	0.0023	3.02E-08	-0.0076	0.031	0.8055
rs3093888	-0.029	0.0045	1.52E-10	0.0302	0.0199	0.1302
rs35446936	-0.094	0.0023	3.56E-198	-0.0211	0.078	0.7865
rs35640778	-0.209	0.007	9.57E-195	0.0017	0.0213	0.9363
rs3767952	0.013	0.0024	1.80E-08	-0.0074	0.0218	0.733
rs3785074	0.024	0.0022	2.64E-27	0.0418	0.0206	0.042
rs3891167	-0.043	0.0024	1.20E-70	0.0112	0.0464	0.8087
rs41304832	0.061	0.0093	5.01E-11	-0.0113	0.0236	0.6338
rs429358	0.017	0.0028	3.82E-10	0.0146	0.0176	0.4067
rs4498805	0.015	0.002	5.65E-14	-0.0166	0.0181	0.3608
rs4530278	0.014	0.0021	1.50E-11	0.0177	0.0182	0.3304
rs45604339	-0.02	0.0021	4.29E-22	-0.0413	0.0181	0.0225
rs4616688	-0.017	0.002	4.51E-18	-0.001	0.0177	0.9528
rs4695407	0.014	0.002	1.46E-12	-0.0714	0.0287	0.0128
rs4724	-0.055	0.0031	9.81E-69	-0.0075	0.0214	0.7257
rs4731541	0.015	0.0024	5.14E-10	0.0435	0.0386	0.2597
rs55747751	-0.021	0.0038	1.70E-08	0.0298	0.021	0.1567
rs56799554	-0.026	0.0027	3.05E-22	-0.0415	0.018	0.0207
rs5742915	0.019	0.002	1.55E-21	0.0045	0.0219	0.8371
rs59409453	0.02	0.0023	1.61E-18	-0.0321	0.0185	0.0834
rs6007020	0.014	0.0021	4.77E-12	0.0016	0.0223	0.9417
rs6054257	-0.014	0.0025	1.07E-08	-0.0043	0.0992	0.9658
rs61405042	-0.05	0.006	8.54E-17	-0.0958	0.039	0.0141
rs61748181	-0.059	0.006	2.79E-23	-0.0286	0.0228	0.2092
rs6536702	0.053	0.0024	9.44E-111	0.0033	0.0189	0.8601
rs6584579	0.011	0.002	1.97E-08	0.0685	0.0246	0.0054
rs6587577	-0.018	0.0026	4.84E-12	0.0365	0.0179	0.0415
rs6659669	-0.012	0.0021	1.15E-08	-0.0106	0.0178	0.5522
rs6669563	0.018	0.002	2.13E-19	0.0359	0.0224	0.108
rs66731853	-0.018	0.0022	1.54E-16	-0.0139	0.023	0.5446
rs6751209	-0.014	0.0025	1.57E-08	0.0313	0.0179	0.0799
rs6776756	-0.017	0.002	1.11E-17	-0.0442	0.021	0.0356
rs6790988	0.015	0.0023	1.78E-10	0.0033	0.0198	0.8699
rs6881568	0.017	0.0021	3.71E-16	0.0237	0.0233	0.3086
rs7099229	-0.015	0.0022	8.44E-12	0.0281	0.018	0.1193
rs7164950	0.013	0.002	2.28E-10	-0.0337	0.0178	0.0586
rs7209057	0.012	0.002	5.68E-09	0.0018	0.0197	0.9256
rs7221585	0.014	0.0025	6.65E-09	-0.0285	0.0322	0.3768
rs73581419	0.023	0.0032	1.34E-12	-0.0722	0.0354	0.0413

rs73730598	0.027	0.0044	4.69E-10	0.012	0.0231	0.6018
rs76065543	0.034	0.0029	4.22E-32	-0.1296	0.0488	0.0079
rs76219171	0.036	0.0043	7.78E-17	0.0155	0.0258	0.5473
rs76666449	0.03	0.0033	8.17E-19	-0.0201	0.019	0.2911
rs7705526	0.078	0.0022	2.43E-282	0.0001	0.0563	0.998
rs7772289	0.018	0.002	1.72E-18	-0.0224	0.0182	0.218
rs77732866	0.018	0.0029	9.16E-10	0.0478	0.0255	0.0605
rs7790856	-0.044	0.0022	1.80E-87	0.0265	0.0213	0.2128
rs78491606	-0.076	0.0074	1.90E-24	0.0284	0.0546	0.6028
rs79977579	0.028	0.0034	2.34E-16	-0.0373	0.0303	0.2189
rs80116508	-0.035	0.0042	1.98E-17	-0.0145	0.0326	0.6558
rs80324517	0.04	0.0047	1.84E-17	-0.0135	0.0363	0.7096
rs8102497	-0.015	0.002	1.40E-13	-0.0386	0.0178	0.0304
rs8105767	0.033	0.0022	2.49E-50	0.018	0.019	0.3437
rs869785	-0.015	0.0021	4.45E-12	0.0117	0.0187	0.5299
rs871134	-0.018	0.002	1.71E-19	0.0322	0.0178	0.0704
rs932002	-0.04	0.0028	7.31E-47	0.0268	0.0209	0.198
rs9398196	-0.014	0.002	9.51E-13	0.0037	0.0183	0.8419
rs939916	0.024	0.0022	6.63E-29	0.0094	0.02	0.638
rs9419958	-0.081	0.0029	2.64E-167	0.064	0.0277	0.021
rs9600019	0.013	0.0021	2.43E-09	-0.0017	0.0183	0.9278
rs9878436	-0.014	0.002	1.20E-12	0.0022	0.0185	0.9048
rs9940099	-0.034	0.0041	3.21E-16	-0.0143	0.0325	0.6604
rs9955360	-0.019	0.003	2.18E-10	-0.049	0.0273	0.0713

SNP, single-nucleotide polymorphism; TL, telomere length ; CAVS, calcific aortic valvular stenosis; Beta, the regression coefficient based on the telomere length effect allele; SE, standard error
